# Setting health research priorities using the CHNRI method: VII. A review of the first 50 applications of the CHNRI method

**DOI:** 10.7189/jogh.07.011004

**Published:** 2017-06

**Authors:** Igor Rudan, Sachiyo Yoshida, Kit Yee Chan, Devi Sridhar, Kerri Wazny, Harish Nair, Aziz Sheikh, Mark Tomlinson, Joy E Lawn, Zulfiqar A Bhutta, Rajiv Bahl, Mickey Chopra, Harry Campbell, Shams El Arifeen, Robert E Black, Simon Cousens

**Affiliations:** 1 Centre for Global Health Research, The Usher Institute for Population Health Sciences and Informatics, The University of Edinburgh, Scotland, UK; 2 Department for Maternal, Newborn, Child and Adolescent Health, World Health Organization, Geneva, Switzerland; 3 Nossal Institute for Global Health, University of Melbourne, Victoria, Australia; 4 Centre for Medical Informatics, The Usher Institute for Population Health Sciences and Informatics, The University of Edinburgh, Scotland, UK; 5 NRF Centre of Excellence in Human Development, DVC Research Office, University of Witwatersrand, Johannesburg, South Africa; 6 Department of Psychology, Stellenbosch University, Stellenbosch, South Africa; 7 Centre for Maternal, Adolescent, Reproductive and Child Health, London School of Hygiene and Tropical Medicine, Keppel Street, London, United Kingdom; 8 Centre for Global Child Health, the Hospital for Sick Children, Toronto, Canada; 9 Centre of Excellence in Women and Child Health, the Aga Khan University Karachi, Pakistan; 10 The World Bank, Washington, DC, USA; 11 Child Health Research Foundation, Dhaka Shishu Hospital, Dhaka, Bangladesh; 12 International Centre for Diarrhoeal Disease Research, Bangladesh, Dhaka, Bangladesh; 13 Institute for International Programs, Johns Hopkins Bloomberg School of Public Health, Baltimore, Maryland, USA; 14 Department of Infectious Disease Epidemiology, London School of Hygiene and Tropical Medicine, London, UK

## Abstract

**Background:**

Several recent reviews of the methods used to set research priorities have identified the CHNRI method (acronym derived from the “Child Health and Nutrition Research Initiative”) as an approach that clearly became popular and widely used over the past decade. In this paper we review the first 50 examples of application of the CHNRI method, published between 2007 and 2016, and summarize the most important messages that emerged from those experiences.

**Methods:**

We conducted a literature review to identify the first 50 examples of application of the CHNRI method in chronological order. We searched Google Scholar, PubMed and so–called grey literature.

**Results:**

Initially, between 2007 and 2011, the CHNRI method was mainly used for setting research priorities to address global child health issues, although the first cases of application outside this field (eg, mental health, disabilities and zoonoses) were also recorded. Since 2012 the CHNRI method was used more widely, expanding into the topics such as adolescent health, dementia, national health policy and education. The majority of the exercises were focused on issues that were only relevant to low– and middle–income countries, and national–level applications are on the rise. The first CHNRI–based articles adhered to the five recommended priority–setting criteria, but by 2016 more than two–thirds of all conducted exercises departed from recommendations, modifying the CHNRI method to suit each particular exercise. This was done not only by changing the number of criteria used, but also by introducing some entirely new criteria (eg, “low cost”, “sustainability”, “acceptability”, “feasibility”, “relevance” and others).

**Conclusions:**

The popularity of the CHNRI method in setting health research priorities can be attributed to several key conceptual advances that have addressed common concerns. The method is systematic in nature, offering an acceptable framework for handling many research questions. It is also transparent and replicable, because it clearly defines the context and priority–setting criteria. It is democratic, as it relies on “crowd–sourcing”. It is inclusive, fostering “ownership” of the results by ensuring that various groups invest in the process. It is very flexible and adjustable to many different contexts and needs. Finally, it is simple and relatively inexpensive to conduct, which we believe is one of the main reasons for its uptake by many groups globally, particularly those in low– and middle–income countries.

The global health research system is an extremely complex network of many diverse actors. It includes large funding agencies, national, regional and international organizations, pharmaceutical and biotech industry and philanthropy–oriented foundations, all of which invest in health research with different aims [1]. The research itself thrives in well–managed and meritocratic universities and research institutes, but also in the private sector. It is assisted by life–long education opportunities for scientists, the supporting industries that develop new research tools, and even by “citizen scientists” – a new breed of researchers [2]. Scrutiny over the health research process is in the hands of many individual research policy makers, ethics committees, peer reviewers of grant proposals and research articles. The dissemination and translation of the results is in the hands of governments, professional bodies, publishers and journal editors, conference organizers, guidelines developers, but also science–focused journalists and media, patent lawyers and many other stakeholders [1]. All of these individuals, groups and organizations act together continuously to conduct, facilitate, support and promote health research and utilize its results. Their collective aim is to generate new knowledge on human health and disease and improve health outcomes for our planet's population [2].

Given that a spectrum of possible ideas for health research is extremely broad and diverse, a need to prioritize between competing research questions arises at different levels – globally, regionally, nationally and locally. Therefore, the process for setting health research priorities is a genuine need and it is being exercised in various forms, but the effectiveness of different approaches is very difficult to evaluate. A recent review described and compared priority–setting tools used in health research prioritization in the 21st century [3]. There seems to be a general consensus among researchers that a flexible, systematic, transparent and replicable process for setting health research priorities would be a desirable tool that could improve the legitimacy of priority–setting exercises at all levels [3,4].

## The CHNRI method for setting health research priorities

The Child Health and Nutrition Research Initiative (CHNRI) started as an initiative of the Global Forum for Health Research in Geneva, Switzerland [4]. One of its main aims was to develop a tool that could assist decision–making and priority setting in health research investments to improve child health and nutrition. Their method also sought to achieve an acceptable balance between fundamental research, translational research and implementation research in order to maximize the potential of health research in reducing both disease burden and the inequities among the world's children [5].

The CHNRI method was developed between 2005 and 2007 through 12 consecutive meetings of a trans–disciplinary panel of 15 experts, supported with funding from the World Bank. The experts worked together to address a number of key challenges related to the multi–dimensional problem of setting priorities in health research investments [5–7]. The method aimed to carefully define the *context* for health research priority setting. The components of the context were: (i) the health issue on which the research is focused; (ii) the affected population that would benefit from the investments in health research; (iii) the timeframe within which the impact of supported research was expected (eg, short, medium or long term); (iv) the style of investment (eg, risk aversive or risk–seeking); and (v) the expected returns from investment (eg, burden reduction, patents, or various forms of public recognition) [6–8].

The method also introduced a systematic approach to listing many competing research questions. It identified four fundamental instruments of health research – “*the four D’s*” – research to achieve (i) *description* (through epidemiological research), (ii) *discovery* (through basic, ie, fundamental research), (iii) *development* (through translational research) and (iv) *delivery* (through health policy and systems research, which includes operations and implementation research). Moreover, it addressed the difference in depth and breadth of suggested research questions by categorizing them in broad *research avenues*, more focused *research options* (which correspond to a 5–year research program), and very specific *research ideas/questions* (which correspond to a typical research article). Finally, the method introduced a transparent *set of criteria* that could discriminate between many competing research options. CHNRI’s “standard” set of criteria followed a simple conceptual framework that demonstrated how the process of health research generates new knowledge. The five suggested criteria were (i) answerability, (ii) effectiveness, (iii) deliverability, (iv) the potential for a substantial reduction of disease burden and (v) the impact on equity [6–8].

The typical CHNRI process involves a small management team that reaches out to a large number of researchers (but also policy–makers and program managers, depending on focus of the exercise) who contribute hundreds of research ideas [9,10]. Once a list of a manageable number of research ideas/questions (usually up to 200) is consolidated by removing overlapping ideas and integrating related ideas, a number of researchers (from 20 to up to several hundreds, depending on the context) are invited to score all proposed research questions against each priority–setting criterion [7,10]. Their input measures “collective optimism” on a scale 0–100. In the final step, external stakeholders are invited to set different thresholds and weights for each of the priority–setting criteria, giving some criteria greater importance over the others, so that the overall score also includes the value system of a wider community [2]. The final output of the CHNRI process is a list that ranks up to 200 research ideas/questions by their scores against several transparent priority–setting criteria [7]. This serves to reveal strengths and weaknesses of all submitted research questions to the research community, judged by a subset of this community using several key criteria for prioritization [8].

## The examples of implementation

We conducted a review of the literature to identify the first 50 examples of the application of the CHNRI method in chronological order, to study the evolution of the uptake of the method. There are presently more than 50 examples of application, with further CHNRI exercises being conducted or planned, but not all of them have reached their final stage of peer–reviewed publication. Therefore, to acknowledge a milestone in method's implementation, we decided to focus on the first 50 publications that have been reviewed and published. We searched Google Scholar, PubMed and so–called “grey literature” (usually defined as papers produced by organizations outside of the traditional publishing and distribution channels) using the search term “CHNRI” or “Child Health and Nutrition Research Initiative”. The first 50 CHNRI priority–setting exercises, published between 2007 and 2016 (the full list with details of each study is available in Table S1 in **Online Supplementary Document(Online Supplementary Document)**), reached out to nearly 5000 researchers, policy makers and program officers, seeking their participation in the generation of research ideas/questions and the scoring of those questions according to the proposed criteria. The initial response rate across all exercises was above 60%, with more than 3000 experts submitting research ideas. They submitted about 10 000 ideas (more than 3 per expert). The redundancy rate in submitted questions was slightly above 50%, indicating a relatively high rate of duplicate ideas. Eventually, 4282 ideas were scored (an average of 86 per exercise) by 2403 participating scorers (an average of 48 per exercise). Most of the papers were published in journals including *PLoS Medicine* (20%), *BMC Public Health* (14%) and *Lancet* (12%). Among the six exercises published in the *The Lancet* journal, three were published as stand–alone exercises and three were a part of policy recommendation papers or “calls for action” within the Lancet series (see Table S1 in **Online Supplementary Document(Online Supplementary Document)**).

Clarity over the context of prioritization and the criteria used for prioritization is one of the key conceptual advances of the CHNRI method. Given the history of the development of the CHNRI method and its initial focus on the reduction of child mortality, it is not surprising that the majority of the exercises have addressed child mortality (either all–cause or specific causes) (52%) (**Table 1**). The use of the CHNRI method was then extended to questions related to childhood morbidity and improved development (4%). In a logical progression of the method's application to address the key global health issues, it was applied to questions of maternal, perinatal and sexual health (8%), followed by several major infectious diseases, such as tuberculosis and zoonoses (6%). Then, the method started to find its application in areas outside of its initial focus – such as mental health (16%), all–cause mortality, morbidity and disability in adults (8%) and dementia (2%). Most exercises were focused on low– and middle–income countries (50%). Further 32% of CHNRI exercises were global in scope, but there were also 14% of exercises conducted at the national level, and 2% at a sub–national level (**Table 1**). This shows that application of the CHNRI method is beginning to expand to health issues beyond the initial focus on child health, and to national and sub–national levels, where there is also a lot of need for prioritization of health research. This is further reflected in 56% of exercises being focused on children (including newborns), 16% on adolescent and young adults, and 28% on adults or all age groups (**Table 1**).

**Table 1:** The main characteristics of the design of the 50 research priority–setting exercises based on the CHNRI method published to date related to the context of the exercise

Health issue addressed through research	Number	Proportion (%)
Child mortality (all–cause or individual causes)	26	52
Child morbidity and suboptimal development	2	4
Sexual health	4	8
Major infectious diseases (eg, tuberculosis, zoonoses)	3	6
All–cause disability	1	2
Mental health	8	16
Dementia	1	2
Health and education system related research	2	4
All–cause morbidity and mortality	3	6
**Context of the CHNRI exercise:**		
Global	16	32
Low– and middle–income countries:	25	50
National	7	14
Sub–national	1	2
Crisis setting	1	2
**Time frame until the expected impact of research:**		
Less than 10 years	10	20
10 years	37	74
More than 10 years	3	6
**Population that would benefit from research:**		
Stillbirths or neonates (<1 month)	7	14
Children aged 1 month – 5 years	17	34
Children older than 5 years	4	8
Adolescents and young adults	8	16
Population aged 60 and above	1	2
People with HIV / with mental health illnesses / disability	4	8
All age groups	9	18
Involvement of external stakeholders:*		
Yes	13	26
No	37	74

*Population groups other than funders of research and their representatives, researchers and/or technical experts involved in the exercise.

In terms of the adopted time frame until the expected impact of research, the large majority of the exercises (74%) used a “standard” time frame of 10 years, originally suggested in the guidelines for implementation of the CHNRI method. A sizable minority of the exercises deviated from the recommended timeframe to suit the contexts to which the exercises were conducted; 20% of the exercises had shorter timeframes, while 6% had longer time frames (**Table 1**). The evolution of the originally proposed CHNRI method through its implementations is particularly apparent when the criteria used for prioritization are analyzed across the 50 exercises. The originally proposed 5 criteria were used only in one–third of the exercises, while they were modified in two–thirds. Modification included changes in the number of criteria used, and the changes in the criteria themselves. Although 56% of all exercises used 5 criteria, as originally suggested, 12% reduced their number to only four or three, while 32% expanded the number of criteria applied – up to 13 in one exercise. Interestingly, although the five “standard” criteria were used most frequently, as expected (from 86% for equity to 70% for effectiveness), it is clear that the groups conducting the CHNRI processes felt a need to replace them and/or introduce further criteria in their exercises, or even reduce their number. The most frequently added criteria were feasibility (in 22% of all exercises), acceptability (22%), low cost (22%), sustainability (22%) and relevance (12%). This shows the flexibility of the CHNRI process in allowing the use of different priority–setting criteria. Adjustments of the process to the needs of each specific exercise should be strongly encouraged (**Table 2**).

**Table 2:** The main characteristics of the design of the 50 research priority–setting exercises based on the CHNRI method published to date related to the criteria used for prioritization

	Number	Proportion (%)
Number of priority–setting criteria used:*		
Three	2	4
Four	4	8
Five	28	56
Six	5	10
Seven or more	11	22
**Priority–setting criteria most frequently used:**		
Equity	43	86
Answerability	42	84
Impact on disease/disability burden	39	78
Deliverability	36	72
Effectiveness	35	70
Low cost	11	22
Sustainability	11	22
Acceptability	11	22
Feasibility	11	22
Relevance	6	12
Applicability	4	8
Ethical	3	6
Attractiveness and originality	3	6
Fundability	2	4
Fills a key gap / potential for breakthrough	2	4
Clarity	2	4
Potential for translation	2	4
Local ownership	2	4
Usefulness (eg, for guiding policies and programmes)	2	4
Sensitivity/immediacy/long–term impact/obstacles to scale–up/need/quality/operationalizability	1	2

*Less than a third (n = 16) of all exercises used the original, “standard” set of the CHNRI criteria; more than two–thirds (n = 34) of the exercises modified the set to adjust it to the need of a particular exercise.

## The main messages from the conducted exercises

As a whole, the 50 CHNRI exercises generated several very broad messages relevant for health research policy. First, if the health issue that was the focus of the prioritization exercise was not well understood in terms of its burden in the population, or the risk factors that contributed to the issue, or the interventions that could be effective in controlling and mitigating the issue, then descriptive (epidemiological) research was identified as the leading research priority as a rule. This showed that generating the knowledge on the burden of the health issue and its “architecture” (in terms of contributing factors and effective interventions) was usually identified as the leading research priority, wherever such knowledge was unavailable.

Given that most contemporary health issues have a reasonably well–defined burden in the population and risk factors, and that effective interventions to reduce or control the burden do exist but are not being implemented, it is not surprising that research on delivery, including health policy and systems, along with operations and/or implementation research frequently dominated the exercise, particularly in low– and middle–income countries [11]. An additional important factor that explains why delivery research was frequently identified as a research priority is the relatively short time frame within which the impact was expected in most exercises (eg, 10 years) and greater urgency to reduce child mortality among the under–privileged populations of the world. Had the health issue been less devastating (eg, mild chronic diseases), and the specified time frame longer (eg, 20–30 years), it is very likely that research priorities would have shifted toward development research and discovery research [11].

Still, there were many examples where “*development*” (translational) research questions and “*discovery*” (basic, ie, fundamental) research questions made it close to the top of the list of priorities. Translational research questions were scored highly wherever there were pre–existing and effective interventions which required some clearly defined and straight–forward modification so as to enable their scale–up in low– and middle–income settings (eg, vaccines stable at high external temperatures). Research questions that required discovery (fundamental) research were prioritised in the exercises where the time frame was longer than 10 years and where hardly any effective interventions were available to reduce or control the health issue (eg, the effect of exercise on dementia and Alzheimer disease [12]). This begs the questions: 1) what time horizon(s) grant agencies adopt and how these differ across agencies; and 2) whether this is explicit or implicit and how this is decided – as the time frame of research questions clearly influences research prioritisation.

## The key advantages of the CHNRI method

We believe that the popularity of the CHNRI method in setting health research priorities can be attributed to several key advances that it proposed. These advances addressed common concerns that persisted following the previous exercises. First, the CHNRI method is *systematic,* because it offered an acceptable framework for handling an endless spectrum of research questions, which provided equal opportunity to questions from different categories of health research.

Second, it is also *transparent,* because it clearly defines the context and priority–setting criteria and provides a replicable approach. All stages of the process and all input can be easily documented and stored in the form of a numerical data set upon which the priorities can be set.

Third, the CHNRI process is *democratic*. It relies on a “crowd–sourcing” approach to both submission of research questions and scoring of the proposed questions. In this way, no single participant in the exercise can have a decisive (or undue) influence on the final ranks. The scores reflect the *collective opinion* of the sample of researchers and other experts from the research community, with each individual input contributing only a minor fraction to the overall scores. The central idea of the crowd–sourcing principle is that a diverse collection of independently–deciding individuals will be likely to make certain types of decisions and predictions better than any experts in the great majority of cases [13].

Fourth, the CHNRI process is *inclusive*, fostering “ownership” of the results by ensuring the various groups invest in the process. This means that an appropriate role is given to donors, researchers and other stakeholders, all of whom can have a substantial influence on the final list of priorities: donors, through defining the context and criteria [9]; researchers, through providing research questions and scoring them [10]; and other stakeholders, through being able to assign more importance (weight) to some criteria over the others [2].

Fifth, the CHNRI process is extremely *flexible* and adjustable to many different contexts and needs. It is very easy to modify it by adjusting the components of the context and adding additional useful priority–setting criteria, as demonstrated through these first 50 applications. Sixth, the CHNRI process is extremely *simple*, which we believe is one of the main reasons for its uptake by many groups globally that haven't been trained in the application of the method. It is enough to study any previously conducted exercise to be able to easily organize and conduct it within any other setting. Although quantitative in its outcomes, the CHNRI method is based on a simple, qualitative input (Yes/No), avoiding any complicated mathematical or statistical computation to obtain the results. Intuitive scores that range between 0–100% and measure collective optimism of a group of experts toward each component of each research question are understandable to users, replicable, amenable to agreement statistics, post–exercise validation and evaluation [14,15]. Seventh, the CHNRI method is reasonably *inexpensive* to conduct. Finally, the results of the CHNRI method are relatively easy to disseminate to the global audience, as the process for priority–setting is structured, objective, replicable and transparent.

## The main points of concern to address in the future

There are several concerns that were expressed in relation to the CHNRI process and they will need careful addressing. First, there is a risk that the spectrum of research ideas submitted and evaluated in the CHNRI process is not comprehensive and that it is missing some particularly promising research questions. Second, the response rate of the invited researchers, policy–makers and program leaders typically ranges between 30–70%, which means that a significant response bias could be introduced at this step [9]. It should be explored whether those who responded to the invitation to participate in the CHNRI exercises differed significantly from those who declined [9]. Third, statistical simulations using data sets from the conducted CHNRI exercises established the minimum number of expert scorers required per exercise to achieve “stable” scores and ranks, above which further addition of experts is unlikely to change the results, and these thresholds should be respected [14,15]. Fourth, a series of experiments on quantitative properties of human collective knowledge and opinion was designed and conducted to demonstrate that collective predictions indeed out–perform individual predictions in the vast majority of cases, but there were still some individuals who managed to out–perform the group's prediction [14,15].

Another risk of bias comes from the process of compiling and combining research questions. Reducing several hundreds of research ideas/questions to a number that is feasible for scoring, such as 200 or less, is an important step. It requires knowledge of the subject matter and is therefore usually performed by a very small group of process managers. The way questions are phrased, or how broadly they are framed, may influence the responses and could introduce bias at this step.

Ultimately, it should be demonstrated that the publications based on the CHNRI process have at least some impact on health research funders and research communities. This could be achieved through analysis of bibliometric indicators, showing the impact of the CHNRI papers on the research community and comparing the intensity of research on identified priorities before and after each of the exercises was published. More importantly, a series of interviews with research policy makers at key funding institutions should be conducted to learn whether they are aware of the CHNRI method and if they have been using it themselves to set research priorities, or used the results of the conducted exercises in their decision–making.

## Opportunities for further development and implementation

The CHNRI method for setting health research priorities was developed to support decision–making for investments in international child health research at a regional level (low– and middle–income countries). However, its advantages have helped its expansion beyond its initial boundaries. There are clearly many opportunities to implement the CHNRI method to address research priorities relevant to all other population health issues. Moreover, the ease of implementation and low cost should help its implementation at a global, national and sub–national level. The development of a massive open online course (MOOC) in CHNRI implementation may facilitate its wider adoption. Another welcome progress would be the development of a free web–based and mobile phone–based and fully automated CHNRI application platform, which would further simplify the exercise and the computation of scores and agreement statistics, based on widely available spreadsheet software.

Finally, the CHNRI method shows how the area of global health may be particularly receptive to solutions based on “the wisdom of crowds” and crowd–sourcing. The CHNRI exercise could be conducted to set priorities among further ideas for crowd–sourcing–based solutions in global health. The world–wide web, mobile phones and crowd–sourcing could potentially serve to generate a massive amount of useful information in real time and solve a diverse set of problems ranging from coordinating funding support, alerting the development of epidemics, identifying areas of medical supplies shortage, monitoring program implementation over large geographic areas, estimating disease burden, effects of risk factors and impact of implemented health interventions in real–time, and many others.

## CONCLUSIONS

Major investment decisions are continuously being made by a variety of funding agencies, but the processes of decision–making and priority setting are rarely systematic and fully transparent. The CHNRI method was developed specifically to address this need. A decade of experience with applying the CHNRI method across a range of contexts and domains has shown that the method is widely acceptable, transparent and replicable. We believe that it has the potential to be scaled up, especially at the national level, and to address health problems outside of child health and nutrition. To encourage its wider use, we will be developing a number of support tools to facilitate its implementation by international, regional, national and local funding agencies.

In the coming years, it will be useful to explore whether the results of the CHNRI method's application, which mainly focused on the context defined by the Millennium Development Goals, would remain relevant to the period until 2030. We will need to explore whether the research ideas/questions identified as priorities remain valid beyond 2015, or do some of the CHNRI exercises need to be repeated with new targets and time horizons? Finally, with an increasing number of the CHNRI exercises being published, and different areas of health research addressed, it should be interesting to explore whether there is an integrated set of priority questions, eg, around implementation models or integration of health system, that can be particularly highlighted as important across most of the conducted exercises? It also remains to be seen whether, as a collective and assisted with modern technology, we could indeed achieve far more to improve global health and development, than we managed to achieve historically through the activities of highly motivated champions.
