# Supplementary material for: Setting health research priorities using the CHNRI method: VII. A review of the first 50 applications of the CHNRI method
Source: J Glob Health. 2017 Jun 18;7(1):011004. doi: 10.7189/jogh.07.011004 (PMC5481891; doi:10.7189/jogh.07.011004)
Supplement: Online Supplementary Document [file jogh-07-011004-s001.pdf]

Online Supplementary Document

Rudan et al. Setting health research priorities using the CHNRI method: VII. A review of the first 50 applications of the CHNRI method

J Glob Health 2017;7:011004

Table S1. Details of the first 50 CHNRI exercises conducted to set research priorities. (Glo = global; Reg = regional; Nat = national; LMIC = low and middle income countries;).

N	Topic	Author	Journal	Year	Reference	Level (Glo/Reg /Nat)	Time frame	Population	Target burden	Criteria	Modifications (explained)	Experts appro-ached	Ideas (sub-mitted)	Ideas (consi-dered)	Sco-rers	Stake-holders
1	Child mortality in South Africa	Tomlinson M et al.	PLoS Med	2007	4(8):e259	National (S. Africa)	10 years	Children (0-4 yrs)	Child mortality (all cause)	5	No	6	63	63	6	Yes (30)
2	Services for mental disorders	Chisholm D et al.	Lancet	2007	370:1241-52	Global	10 years	Persons with one of four major mental health issues	Schizophrenia; Depression; Substance abuse and alcoholism; Adolescent and children's disorders	5	No	24	100	100	24	No
3	Primary health care	Walley J et al.	Lancet	2008	372:1001-1007	LMICs	20 years	All age groups	NCDs and maternal & child health	6	Yes ("likelihood of Effectiveness" replaced with "feasibility of undertaking"; and added a criterion: "likelihood to fill a crucial gap in knowledge")	27	69	69	20	No
4	Disabilities	Tomlinson M et al.	Lancet	2009	374:1857-62	Global	10 years	Persons with disabilities	Global burden of disability	5	Yes - 3 criteria changed ("answerability" and "equity" retained; other criteria replaced	82	348	83	50	No

											with "likelihood of applicability", "likelihood for obtaining support" and "potential sensitivity"					
5	Impaired mental health	Tomlinson M et al.	Bull WHO	2009	87:438-446	Global	10 years	Persons with one of four major mental health issues	Schizophrenia; Depression; Substance abuse and alcoholism; Adolescent and children's disorders	5	No	39	290	55	39	Yes
6	Zinc interventions	Brown KH et al.	Public Health Nutr	2009	12:389-396	LMICs	10 years	Children (0-4 yrs)	Morbidity and mortality related to Zn definiency	5	No	7	90	31	7	No
7	Diarrhoeal disease	Kosek M et al.	J Health Popul Nutr	2009	27:319-331	Global	10 years	Children (0-4 yrs)	Morbidity and mortality related to diarrhoea	5	No	17	46	46	10	No
8	Neonatal infections	Bahl R et al.	Pediatr Infect Dis J	2009	Suppl 1:S43-8	LMICs	10 years	Newborns (0-1 mths)	Mortality from infections	5	No	20	69	61	13	Yes (orig)
9	Childhood diarrhoea	Fontaine O et al.	PLoS Med	2009	6(3):e100041	LMICs	10 years	Children (0-4 yrs)	Mortality from diarrhoea	5	No	25	154	154	13	Yes (orig)
10	Pneumococcal diseases	Webster J et al.	BMC Publ Hlth	2011	11 Suppl 3:S26.	LMICs	10 years	Children (0-4 yrs)	Morbidity and mortality from S. Pneumoniae	9	Yes (Emerging interventions only, ie, "discovery"; 9 criteria used: the standard five, and the additional: "low development cost"; "sustainability of implementation"; "acceptability to health care providers; and "acceptability to end users")	20	2	2	20	No

11	Staphylococcal diseases	Huda T et al.	BMC Publ Hlth	2011	11 Suppl 3:S27	LMICs	10 years	Children (0-4 yrs)	Morbidity and mortality from S. Aureus	9	Yes (Emerging interventions only, ie, "discovery"; 9 criteria used: the standard five, and the additional: "low development cost"; "sustainability of implementation"; "acceptability to health care providers; and "acceptability to end users")	20	2	2	20	No
12	Oxygen systems for intensive care	Catto AG et al.	BMC Publ Hlth	2011	11 Suppl 3:S28	LMICs	10 years	Children (0-4 yrs)	Mortality from respiratory infections and sepsis	9	Yes (Emerging interventions only, ie, "discovery"; 9 criteria used: the standard five, and the additional: "low development cost"; "sustainability of implementation"; "acceptability to health care providers; and "acceptability to end users")	20	1	1	20	No
13	Meningococcal diseases	Choudhuri et al.	BMC Publ Hlth	2011	11 Suppl 3:S29	LMICs	10 years	Children (0-4 yrs)	Morbidity and mortality from N. Meningitidis	9	Yes (Emerging interventions only, ie, "discovery"; 9 criteria used: the standard five, and the additional: "low development cost"; "sustainability of implementation"; "acceptability to health care providers; and "acceptability to end users")	20	2	2	20	No

14	RSV-associated respiratory infections	Nair H et al.	BMC Publ Hlth	2011	11 Suppl 3:S30	LMICs	10 years	Children (0-4 yrs)	Morbidity and mortality from RSV	9	Yes (Emerging interventions only, ie, "discovery"; 9 criteria used: the standard five, and the additional: "low development cost"; "sustainability of implementation"; "acceptability to health care providers; and "acceptability to end users")	20	3	3	20	No
15	Measles	Higginson D et al.	BMC Publ Hlth	2011	11 Suppl 3:S31	LMICs	10 years	Children (0-4 yrs)	Morbidity and mortality from measles	9	Yes (Emerging interventions only, ie, "discovery"; 9 criteria used: the standard five, and the additional: "low development cost"; "sustainability of implementation"; "acceptability to health care providers; and "acceptability to end users")	20	1	1	20	No
16	Zoonotic diseases in India	Sekar N et al.	PLoS One	2011	6(2):e17120	National (India)	5 years	All age groups	11 major zoonotic diseases	5	Yes (minor)	17	103	103	5	Yes
17	Birth asphyxia	Lawn JE et al.	PLoS Med	2011	8(1):e100389	LMICs	10 years	Newborns (0-1 mths)	Mortality from birth asphyxia	5	No	26	61	61	21	Yes (LK)
18	Childhood pneumonia	Rudan I et al.	PLoS Med	2011	8(9):1001099	LMICs	10 years	Children (0-4 yrs)	Mortality from pneumonia	5	No	45	511	156	45	Yes (LK)
19	Psychosocial support in humanitarian settings	Tol WA et al.	PLoS Med	2011	8(9):e1001096	Global	10 years	All age groups	Mental disorders and psychosocial issues	5	Yes ("Answerability" and "Equity" retained; others replaced with "Significance",	136	733	74	82	No

											"Ethics" and "Applicability"					
20	Implementation for stillbirths and preterm births	George A et al.	PLoS Med	2011	8(1):e1000380	LMICs	5-10 years	Children (0-4 yrs)	Preterm births and stillbirths	5	Yes ("Answerability" and "Burden reduction" retained; Others replaced by "Addresses obstacles to scale-up"; "Likely to attract funding and national policy attention"; and "Local ownership"	85	55	55	29	No
21	Tuberculosis	Lienhardt C et al.	PLoS Med	2011	8(11):e1001135	Global	5 years	All age groups	Tuberculosis	5	Yes (focus on added value)	50	250	250	50	No
22	Stillbirths	Flenady V et al.	Lancet	2011	377: 1703-17	LMICs	10 years	Stillbirths	Stillbirths	5	Yes ("Attractiveness", "Feasibility" and "Relevance" used in some exercises; Answerability and Equity retained)	50	279	279	50	No
23	Impaired mental health	Collins CY et al.	Nature	2011	475:27-30	Global	10 years	All age groups	Mental health	4	Yes ("Ability to reduce disease burden" and "Impact on equity" used; "Immediacy of impact" and "Feasibility" added)	422	1565	164	33	No
24	Children in adversity	Jordans MJD et al.	Soc Sci Med	2011	73:456-466	LMICs	5-10 years	Children (0-9 yrs)	Mental health	3	Yes ("Acceptability", "Feasibility" and "Effect" used, others dropped)	60	47	17	31	No
25	Preterm birth and low birth weight	Bahl R et al.	J Glob Health	2012	2(1):010403	LMICs	10 years	Newborns (0-1 mths)	Preterm birth and low birth weight	5	No	21	82	82	21	Yes (LK)
26	Emerging interventio	Rudan I et al.	J Glob Health	2012	2(1):010304	LMICs	10 years	Children (0-4 yrs)	Childhood pneumonia,	9	Yes (Emerging interventions only,	20	29	29	20	No

	ns for pneumonia, meningitis and flu								meningitis and influenza		ie, "discovery"; 9 criteria used: the standard five, and the additional: "low development cost"; "sustainability of implementation"; "acceptability to health care providers; and "acceptability to end users")					
27	Impaired mental health in Brazil	Gregorio G et al.	Rev Bras Psiq	2012	34:434-439	National (Brazil)	10 years	All age groups	Mental health	5	No	28	110	35	17	No
28	Children at developmental risk in Chile	Arbour MC et al.	J Dev Behav Pediatr	2012	33:666-675	National (Chile)	1 year	Children (5-7 yrs)	Child development assessment	13	Yes (All CHNRI criteria replaced with 13 different criteria to assess the most suitable instrument for the local context, such as Quality, Administration site, Cost, Time, Spanish translation available, priori use in Chile, etc.)	21	22	22	12	No
29	Reproductive health in crisis settings	Morof D et al.	Int J Gynecol Obstet	2012	119 Suppl3:S429	Crisis settings	5-10 years	Women, adolescents and couples of reproductive age	Mortality and severe morbidity among mothers, fetuses, newborns and children	5	Yes (All criteria changed: Need, Feasibility, Operationalizability, Usefulness, Relevance)	68	94	94	16	No
30	Influenza	Nair H et al.	BMC Publ Hlth	2013	11 Suppl 3:S14	LMICs	10 years	Children (0-4 yrs)	Mortality from influenza	9	Yes (Emerging interventions only, ie, "discovery"; 9 criteria used: the standard five, and the additional:	20	1	1	20	No

											"low development cost"; "sustainability of implementation"; "acceptability to health care providers; and "acceptability to end users")					
31	Adolescent sexual and reproductive health needs	Hindin M et al.	Bull WHO	2013	91:10-18	LMICs	10 years	Adolescents	Sexual and reproductive health problems	5	Yes ("Effectiveness" is replaced with "Clarity"; 2-stage approach to define "themes" first, and then questions within each theme)	296	280	280	144	No
32	Childhood diarrhoea	Wazny K et al.	PLoS Med	2013	10(5): e1001446	Global	15 years	Children (0-4 yrs)	Mortality and morbidity from diarrhoea	5	Yes (2-stage approach to define "themes" first, and then questions within each theme)	200	466	466	150	No
33	Pre-conception care	Dean S et al.	PLoS Med	2013	10(9):e1001508	LMICs	10 years	Women, adolescents and couples of reproductive age	Mortality and severe morbidity among mothers, fetuses, newborns and children	6	Yes - 6 criteria (Five standard ones and an additional - "Potential impact on long-term outcomes for women and children"	130	37	37	48	No
34	Emerging interventions for childhood diarrhoea	Bhutta ZA et al.	J Glob Health	2013	3(1):010302	LMICs	10 years	Children (0-4 yrs)	Childhood diarrhoea	9	Yes (Emerging interventions only, ie, "discovery"; 9 criteria used: the standard five, and the additional: "low development cost"; "sustainability of implementation"; "acceptability to health care	12	10	10	12	No

											providers; and "acceptability to end users")				
35	Mental health care	Jordans MJD et al.	BMC Psychiatry	2013	13:e332	National (Nepal)	10 years	All age groups	Mental disorders and psychosocial issues	3	Yes (Criteria used: "Cultural relevance"; "Frequency" (burden); and "Feasibility"	26	13	13	26	No
36	Newborn health	Yoshida S et al.	Lancet	2014	384:e27-e28	LMICs	10-15 Years	Newborns	Mortality and morbidity in newborns	5	No	132	396	205	91	No
37	Maternal and perinatal health	Souza JP et al.	Reprod Health	2014	11:e61	Global	10 years	Pregnant and post-partum women	Maternal and perinatal health	5	No	339	980	190	140	No
38	Family planning	Ali M et al.	Bull WHO	2014	92:93-98	Global	10 years	Reproductive age	Unmet need for family planning	5	Yes ("Answerability" replaced by "Ethically implemented"; 2-stage approach to define "themes" first, and then questions within each theme)	102	55	47	66	No
39	Neonatal survival in humanitarian emergencies	Morof DF et al.	Confl Health	2014	8:e8	Global	5-10 years	Newborns (0-1 mths)	All cause mortality and disability in humanitarian emergencies	4	Yes ("Answerability" and "Equity" retained; other criteria replaced by "Feasibility" and "Relevance")	97	28	28	35	No
40	Developmental and intellectual disabilities and autism	Tomlinson M et al.	J Intellect Disab Res	2014	58(12):1121-1130	Global	5-10 years	All age groups	Intellectual disability and autism	5	Yes ("Answerability" and Equity" were retained; other criteria were replaced by "Feasibility", "Applicability and impact" and "Support within the context"	72	69	69	49	No

41	Integrated community case management	Wazny K et al.	J Glob Health	2014	4(2):020411	LMICs	10 years	Children (0-4 yrs)	Mortality and morbidity from childhood diseases that could be prevented and/or treated through iCCM	4	Yes ("Equity" was dropped, and "Deliverability" replaced by "Overall feasibility"; 2-stage approach to define "themes" first, and then questions within each theme)	133	366	61	75	No
42	PMTCT in 3 African countries	Rollins N et al.	J Acq Imm Def Synd	2014	67(Suppl2):S 108-13	National (Malawi, Nigeria and Zimbabwe)	5-10 years	People living with HIV/AIDS	PMTCT implementation barriers and child mortality	6	Yes (Effectiveness was dropped. Two criteria was added "innovation and originality" and "likely value to policy maker"	191	239	90	191	Yes (40-70/conutry)
43	Maternal and child health services	Li X et al.	Chin Hlth Serv Manag	2014	310(4):244-247	Subnational (W China)	10 years	Mothers and children (0-4 years)	Morbidity and mortality of mothers and children	10	Yes (Standard + Acceptability; Sustainability; Translation potential; Cost; Ethics)	17	24	24	17	Yes (19)
44	Family planning	Campbell S et al.	SFPRF Report	2014	pp. 1-36	Global	10 years	Women of reproductive age	Family planning - maternal and perinatal health	6	Yes (Standard + "Innovation")	80	53+	53	80	No
45	Acute malnutrition in infants	Angood et al.	PLoS Med	2015	12(4):e1001812.	Global	10 years	Infants less than 6 months	Management of acute malnutrition and child mortality	6	Yes (Standard + "Sustainability")	64	60	60	64	Yes
46	Health policy research direction	Yang et al.	Chinese J Hlth Policy	2015	8(1):74-79	National (China)	5 years	All age groups	All cause morbidity and mortality	5	No	33	50	50	29	Yes (20)
47	Drug-resistant TBC	Velayutham et al.	Public Health Action	2015	5(4):222-235	Global	10 years	Children (0-9 yrs)	Drug-resistant tuberculosis	5	Yes ("Max burden reduction" replaced by "Feasibility")	304	89	53	81	No
48	Education in LMICs	Read et al.	Curr Issues Comp Edu	2015	18(1):55-67	LMICs	10 years	School children	Suboptimal education	4	Yes ("System impact", "School impact", "Student impact", "Feasibility")	84	267	89	37	No

49	Adolescent health	Nagata et al.	J Adolesc Health	2016	59:50-60	LMICs	10 years	Adolescents	Eight areas (communicable diseases, injuries, violence, mental health, noncommunicable diseases, nutrition, physical activity, substance use and health policy)	5	Yes ("Clarity", "Answerability", "Importance", "Implementation", "Equity")	450	512	303	160	Yes (LK)
50	Dementia	Shah et al.	Lancet Neurol	2016	15:1285-94	Global	10 years	Very old persons	Dementia	5	Yes ("Potential for success, potential for conceptual breakthrough, potential for translation, equity, disease burden reduction)	740	493	59	154	No
